# Chicken intestinal microbiota modulation of resistance to nephropathogenic infectious bronchitis virus infection through IFN-I

**DOI:** 10.1186/s40168-022-01348-2

**Published:** 2022-10-03

**Authors:** Hai-chang Yin, Zhen-dong Liu, Wei-wei Zhang, Qing-zhu Yang, Tian-fei Yu, Xin-jie Jiang

**Affiliations:** 1 College of Life Science and Agriculture Forestry, Qiqihar University, Qiqihar, 161006 Heilongjiang China; 2 Department of Surgery of Spine and Spinal Cord, Henan Provincial People’s Hospital, Zhengzhou, 450003 Henan China

**Keywords:** SPF chicken, Intestinal microbiota, Nephropathogenic infectious bronchitis virus, IFN, *Lactobacillus*

## Abstract

**Background:**

Mammalian intestinal microbiomes are necessary for antagonizing systemic viral infections. However, very few studies have identified whether poultry commensal bacteria play a crucial role in protecting against systemic viral infections. Nephropathogenic infectious bronchitis virus (IBV) is a pathogenic coronavirus that causes high morbidity and multiorgan infection tropism in chickens.

**Results:**

In this study, we used broad-spectrum oral antibiotics (ABX) to treat specific pathogen free (SPF) chickens to deplete the microbiota before infection with nephropathogenic IBV to analyze the impact of microbiota on IBV infections in vivo. Depletion of the SPF chicken microbiota increases pathogenicity and viral burden following IBV infection. The gnotobiotic chicken infection model further demonstrated that intestinal microbes are resistant to nephropathogenic IBV infection. In addition, ABX-treated chickens showed a severe reduction in macrophage activation, impaired type I IFN production, and IFN-stimulated gene expression in peripheral blood mononuclear cells and the spleen. *Lactobacillus* isolated from SPF chickens could restore microbiota-depleted chicken macrophage activation and the IFNAR-dependent type I IFN response to limit IBV infection. Furthermore, exopolysaccharide metabolites of *Lactobacillus* spp*.* could induce IFN-β.

**Conclusions:**

This study revealed the resistance mechanism of SPF chicken intestinal microbiota to nephropathogenic IBV infection, providing new ideas for preventing and controlling nephropathogenic IBV.

Video abstract

**Supplementary Information:**

The online version contains supplementary material available at 10.1186/s40168-022-01348-2.

## Introduction

Poultry meat is the main source of nutritional protein that is consumed by humans worldwide [1]. As the European Union bans antibiotics as growth promoters and with the concerning spread of antibiotic resistance [2], alternative methods to prevent infection and maintain healthy chicken flocks are urgently needed.

Mammals’ intestinal microbiota can regulate most enteroviruses and some local or systemic infectious viruses [3–6]. Although there are several studies on the intestinal microbiota of mammals, there are few reports on the intestinal microbiota of chickens, and the molecular mechanisms of the interaction of specific commensal bacteria with poultry hosts are not well understood. Recent studies reveal that depletion of intestinal microbiota by treating chickens with broad-spectrum oral antibiotics (ABX) can promote avian influenza virus (AIV) and Marek’s disease virus infections [7, 8], emphasizing the importance of intestinal microbiota in determining chicken immunity against viral diseases.

Infectious bronchitis virus (IBV), a member of the Coronaviridae family, can cause breathing difficulties, kidney urate deposition, secondary infections, and even death in chickens. IBV was first described in the USA in the 1930s [9], and in the 1960s, the first nephrogenic IBV strain was reported in the USA and Australia and subsequently reported worldwide. Nephrotic IBV has become the most prevalent IBV strain in commercial poultry [10, 11]. Because the genome is prone to mutation and recombination events, new genotypes and variant strains continue to appear, resulting in limited immune cross-protection between different strains, making it extremely difficult to control IBV [12–14]. Currently, severe diseases caused by IBV are still a major problem in the global poultry industry and seriously endanger its development. Therefore, finding new ways to prevent and control IBV infection is important. Although studies have found that IBV can change the diversity of intestinal microbiota [15, 16], whether intestinal microbes have a regulatory effect on IBV infection remains unclear.

Type I interferon (IFN-I) production is rapidly induced when host sensor pattern recognition receptors recognize signals from pathogen-associated molecular patterns, such as viral nucleic acids [17]. These induced IFN-Is transduce signals to the nucleus through a cascade of related molecules and rapidly induce the expression of hundreds of IFN-stimulated genes (ISGs) [18, 19]. Due to the ability to promote viral clearance, induce tissue repair, and stimulate a prolonged adaptive immune response against viruses, IFN-I responses constitute the body’s first line of defense against viral infections, including IBV [20, 21]. IFN-I is regulated by the gut microbiota by acting on a variety of immune cells, including natural killer cells, macrophages, and CD^8+^ T cells [5, 22–24]. Abt et al. (2012) found that treatment with ABX reduced the content of mouse gut microbiota, which in turn suppressed the expression of IFN-β and ISGs in macrophages. Ganal et al. (2012) reported that when ABX-treated and germ-free mice were infected with influenza virus, lymphocytic choriomeningitis virus, murine cytomegalovirus, and Sendai virus, both systemic IFN-I production and ISGs were reduced, resulting in increased viral titers or increased susceptibility of mice to the virus. The microbiota constitutively induce the production and basic level of ISG expression in plasmacytoid dendritic cells (pDC) at the type I IFN system locus [25, 26]. Despite accumulating evidence that the microbiota can promote IFN-I responses in multiple mammalian cell types, whether the chicken gut microbiota induce antiviral IFN-I secretion and the cell type of origin are unclear. Identification of unknown, chicken-specific microbiome species that influence type I IFN antiviral responses still needs to be done.

Here, nephropathogenic IBV was used as an infection model to describe the impact of the SPF chicken gut microbiome on the IFN-I of host-related antiviral innate immunity. Depletion of the intestinal microbiota by gavage with an ABX cocktail exacerbates the infection and clinical symptoms of IBV. Furthermore, we show that intestinal microbes resist IBV infection via the gnotobiotic chicken infection model. Consistent with these phenotypes, ABX-treated (ABX) chickens showed a diminished innate immune response, accompanied by less macrophage activation after IBV infection, which is related to the decreased expression of interferon-stimulated genes (ISGs) in the peripheral blood and spleen. In addition, *Lactobacillus* was used to gavage ABX-treated chickens, which enabled them to induce the expression of IFN-I in macrophages through its metabolite exopolysaccharides (EPSs), regaining the ability to resist nephropathogenic IBV infection.

## Results

### Significant depletion of the microbiota in antibiotic-treated SPF chickens

**Fig. 1:**
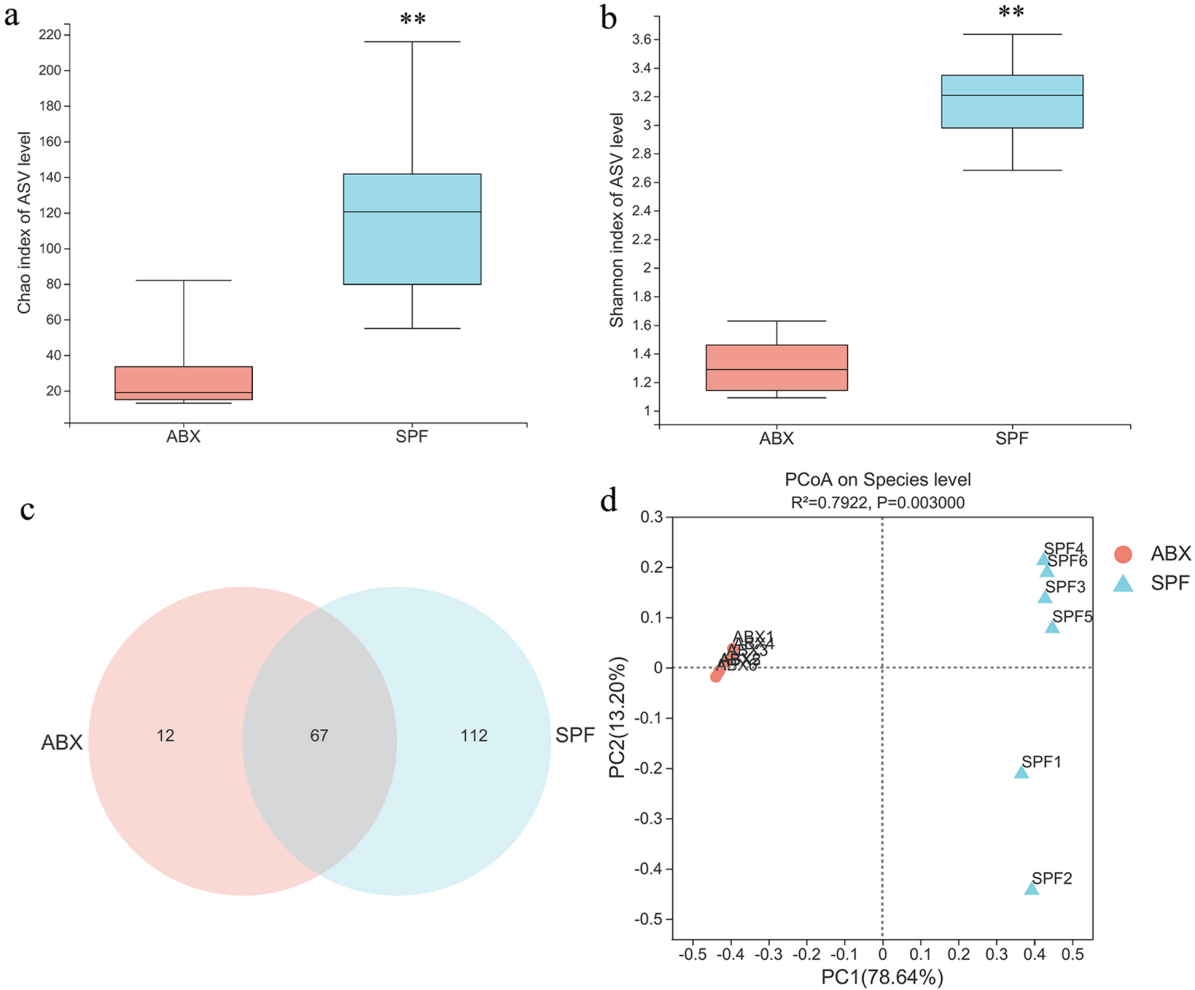
Antibiotic treatment for microbiota depletion. The magnitude by which the ABX (ampicillin, neomycin, metronidazole, and vancomycin) depleted the specific pathogen-free (SPF) chicken (*n* = 6) gut microbiota was assessed by **a** Chao1, **b** Shannon, **c** Venn diagram, and **d** PCoA plot based on unweighted Unifrac analysis. Data are from two independent experiments. Biological replicates for each sample are three. *P* values were determined using unpaired, two-tailed Student’s *t* tests. **P < 0.01

Age- and sex-matched SPF chickens were continuously treated with an ABX cocktail in drinking water. The fresh fecal microbiota of SPF and ABX chickens were analyzed via 16S rRNA sequencing to evaluate the effect of ABX treatment on the intestinal microbiota of SPF chickens. The dilution curve generated based on the operational taxonomic unit (OTU) level indicated that the sampling work had sufficient sequences to analyze bacterial diversity (Additional file 1). The application of ABX resulted in a significant reduction in the abundance (Fig. 1a) and diversity (Fig. 1b) of the intestinal microbiota of the treated chickens. In addition, the Venn diagram showed that, compared with the control group, the number of species in the ABX treatment group was significantly reduced (Fig. 1c). In addition, the principal coordinate analysis (PCoA) diagram based on unweighted UniFrac showed a clear cluster separation between the control and ABX treatment groups (Fig. 1d). These results indicated that ABX treatment resulted in a significant reduction in chicken intestinal microbiota.

### SPF chickens’ microbiota modulation of resistance to nephropathogenic IBV infection

**Fig. 2:**
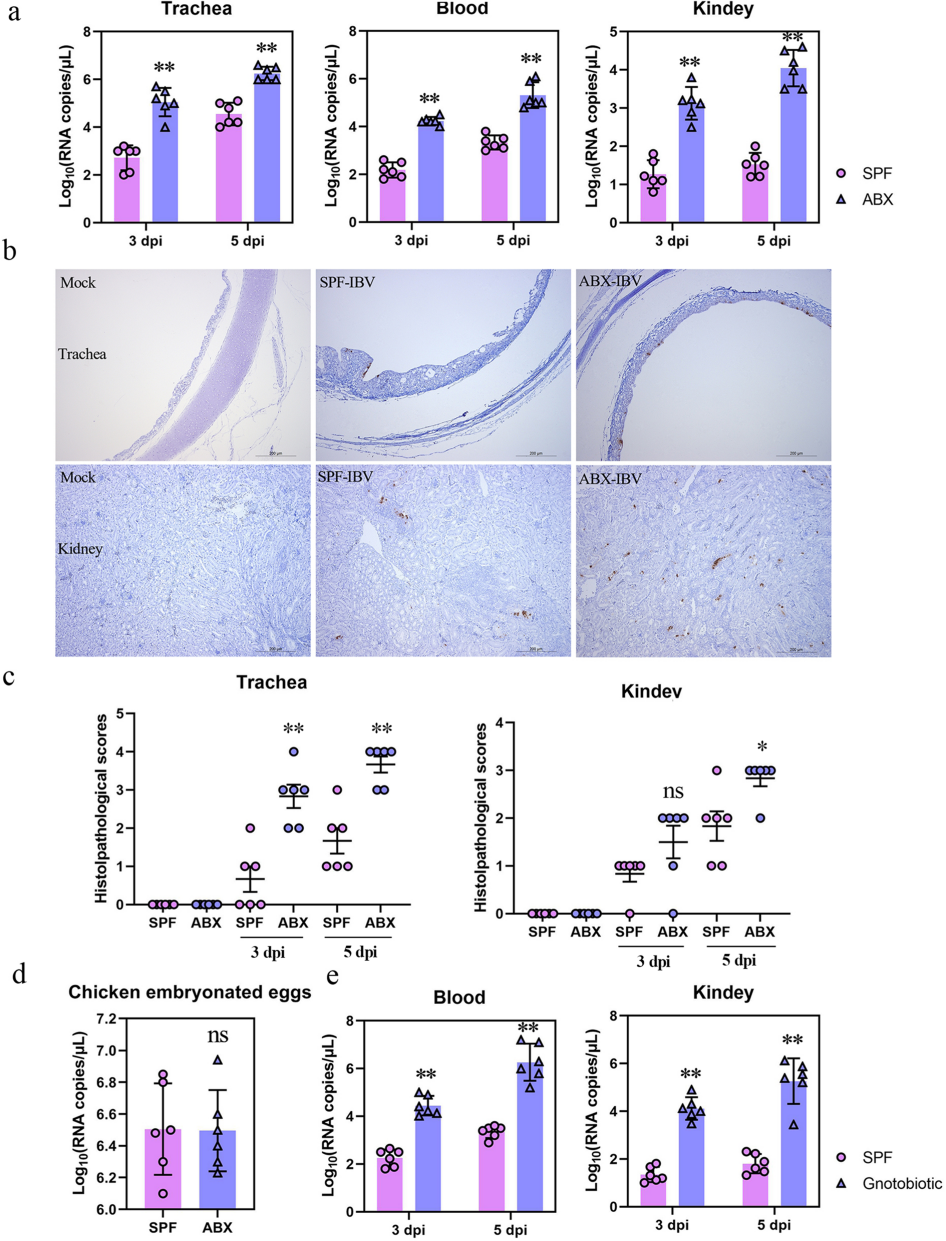
Microbiota depletion accelerated nephropathogenic IBV pathogenesis and viral replication in vivo. **a** Trachea, peripheral blood, and kidney of SPF or ABX chicken were harvested and tested for viral loads at 3 and 5 dpi using qRT-PCR (*n* = 6). **b** Immunohistochemical anti-N staining of hematoxylin-counterstained tracheal or kidney sections (representative images of 4 or 5 per group). Scale bar, 200 μm. **c** Histopathological analysis of IBV infection in SPF and ABX chickens. Histopathological analysis was performed on tracheal or kidney samples from noninfected or IBV-infected chickens (SPF or ABX chickens). Histological scoring of hematoxylin and eosin stain-colored tracheal or kidney sections (*n* = 7 or 8). **d** Consequences of ABX treatment on IBV virus replication in embryonated chicken eggs. Eleven-day-old embryonated chicken eggs (*n* = 6) were injected with ABX or PBS. Viral growth was analyzed by quantifying viral RNA using qRT-PCR from RNA extracted from allantoic fluids collected at 72 h post-infection. **e** Kidney and peripheral blood of gnotobiotic chicken (*n* = 6) were harvested and tested for viral loads at 3 and 5 dpi using qRT-PCR. Data are from three independent experiments. Biological replicates for each sample are three. *P* values were determined by an unpaired two-tailed Student’s *t* test. ns, not significant ; **P < 0.01

SPF chickens administered a cocktail of ABX for 2 weeks were infected with 0.2 mL 10^6^ EID_50_/0.1 mL IBV TW I strain via the oculonasal route. The ABX chickens remained under the same ABX treatment for the entire duration of the experiment. ABX chickens infected with IBV showed various clinical symptoms at 5 days post-infection (dpi), including hunchback and tremor, hind limb paralysis, dyspnea, and death, whereas SPF chickens showed less obvious clinical symptoms, including sneezing and predominant tracheal rales. Quantitative reverse transcription-polymerase chain reaction (qRT-PCR) was used to quantify viral nucleic acids in the trachea, peripheral blood, and kidneys to assess the level of viral replication. The amount of viral nucleic acid in the trachea, peripheral blood, and kidney of ABX chickens was higher than that in SPF chickens at 3 and 5 dpi (Fig. 2a). Consistent with clinical symptoms and viral loads, immunohistochemical analysis showed that at 5 dpi, compared with that in the SPF chickens, there were more viral signals (nucleocapsid N protein) in the tracheal/kidney sections of ABX chickens (Fig. 2b). The results of the histopathological analysis also showed that ABX treatment aggravated the pathological damage caused by IBV to the tissues (Fig. 2c).

SPF chicken embryos (9–11-day-old) were treated with an ABX cocktail to determine whether ABX treatment could directly affect IBV replication independently. One day later, 0.2 mL 10^5^ EID_50_/0.1 mL IBV together with ABX into ABX-treated eggs or PBS was inoculated into SPF chicken embryos. The level of virus replication was assessed using qRT-PCR to quantify viral nucleic acids in allantoic fluid collected at 3 dpi. A similar viral RNA was detected in SPF and ABX chicken embryos (Fig. 2d). Since chicken embryos have almost no bacterial microbiota [27], this result indicated that the increase in IBV replication in chickens observed with ABX treatment was not due to the direct effect of ABX on IBV replication and rather due to the effects mediated by intestinal microbiota.

Owing to the potential off-target effects of antibiotics, a gnotobiotic chicken model was used to confirm the direct effects of commensal microbiota on the observed phenotype. The sterility of the isolator and incubator was determined by culturing feces and swabs from the sterile isolator. All samples were negative for bacterial colonies, indicating that the chickens were germ-free. Compared to that in the SPF chickens, viral replication was detected in the blood and kidneys of gnotobiotic chickens. Consistent with that in ABX chickens, the titers of IBV in the peripheral blood and kidney were increased in gnotobiotic chickens (Fig. 2e).

### Intestinal microbiota induce innate immunity to IBV by activating mononuclear macrophages

**Fig. 3:**
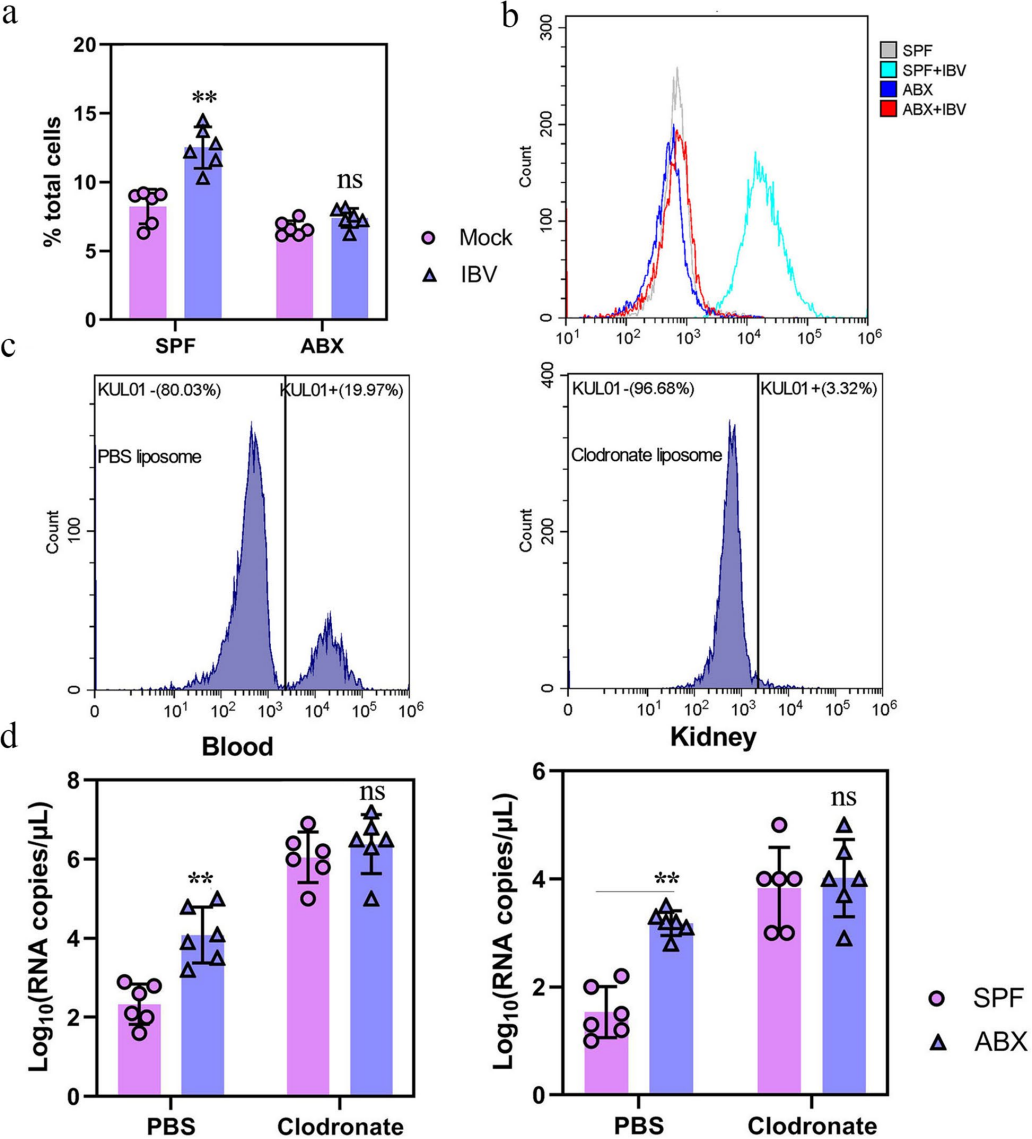
Innate immune responses were diminished in ABX chicken post-IBV infection. **a** Frequency of a panel of macrophages in PBMC of mock or infected SPF or ABX chicken (*n* = 6) at 5 dpi. **b** Expression of MHC-II on PBMC macrophages from mock or infected SPF or ABX chicken (*n* = 6) at 5 dpi. **c** Depletion of splenic macrophages following administration of clodronate liposomes. Histograms showing KUL01+ cells isolated from the spleen of chicken (*n* = 6) treated with PBS liposomes or clodronate liposomes. **d** Blood and kidney viral titers of SPF or ABX chicken (*n* = 6) treated with PBS liposomes or clodronate liposomes at 5 dpi. Data are from three independent experiments. Biological replicates for each sample are three. *P* values were determined by an unpaired two-tailed Student’s *t* test. ns, not significant ; **P < 0.01

Nephropathogenic IBV achieves nephropathogenic hallmarks by actively exploiting mononuclear carrier cells [28]. Therefore, we analyzed whether depletion of SPF chicken microbiota leads to damage in macrophage-related immune responses to IBV infection. First, SPF or ABX chickens were infected with IBV, and monocytes/macrophages were isolated from peripheral blood mononuclear cells (PBMCs) in the peripheral blood at 5 dpi to evaluate macrophage recruitment and activation. The cell extracts were stained with mouse anti-chicken monocyte/macrophage-PE clone KUL01 and quantified using flow cytometry. Compared with that in mock SPF chickens, infected SPF chickens exhibited a significantly increased percentage of macrophages. In contrast, IBV infection did not promote macrophage frequency in infected versus mock-infected ABX chickens (Fig. 3a). Consistent with these findings, we discovered that surface molecules reflecting macrophage activation MHC-II were greatly augmented in the PBMC from infected SPF chickens, however, not in those cells from infected ABX chickens at 5 dpi, compared to that from mock-infected chickens (Fig. 3b). To further verify that SPF chicken intestinal microbiota is necessary for activating macrophages in response to IBV infection, ABX chickens were systemically treated with clodronate liposomes to deplete macrophages and then infected with IBV. The results showed that the depletion of macrophages from the spleen was effective (Fig. 3c). At 5 dpi, IBV titers in the blood or kidney of ABX chickens were higher than those in infected SPF chickens, and no significant difference in viral titers was observed in SPF or ABX chickens after clodronate liposome treatment (Fig. 3d).

### Depletion of SPF chickens’ intestinal microbiota greatly impaired the IFN-I response to IBV infection

**Fig. 4:**
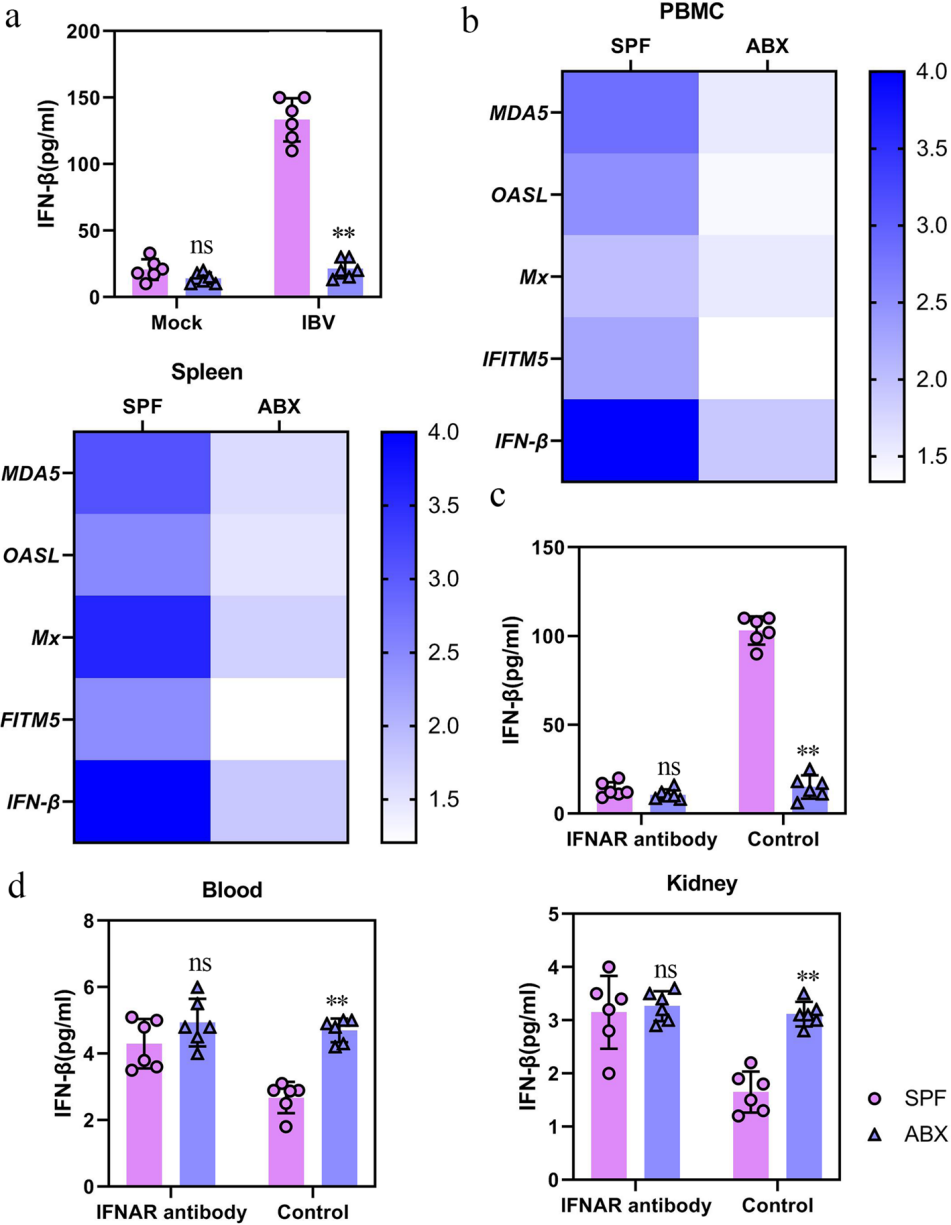
Microbiota depletion results in significantly impaired systemic type I IFN responses to IBV. **a** IFN-β levels in the serum at 5 dpi following IBV infection detected by ELISA (*n* = 6). **b** Fold change of IFN-β and ISG expression in PBMC and spleen of SPF or ABX chicken (*n* = 6) at 5 dpi following IBV infection relative to respective mock controls. **c** IFN-β levels in the serum at 5 dpi from infected IFNAR antibody-treated SPF or ABX chicken (*n* = 6). **d** Viral load in blood or kidney collected from infected IFNAR antibody-treated SPF or ABX chicken (*n* = 6). Data are from three independent experiments. Biological replicates for each sample are three. *P* values were determined by an unpaired two-tailed Student’s *t* test. ns, not significant ; **P < 0.01

As an effector cytokine at the core of host antiviral immunity, the expression of IFN-β is regulated by the intestinal microbiota [29–31]. It is hypothesized that the intestinal microbiota can modulate the IFN-I response in the IBV infection model. ABX and SPF chickens were inoculated with 0.2 mL 10^6^ EID_50_/0.1 mL of IBV. At 5 dpi, the secretion of IFN-β in the serum of chickens was analyzed using an ELISA kit for chicken IFN-β, while mRNA expression of IFN-β and ISGs in PBMCs and spleen were analyzed using qRT-PCR. Compared with that in the serum of SPF chickens, the secretion of IFN-β in the serum of ABX chickens was reduced (Fig. 4a), and the expression of IFN-β and ISGs in PBMC and spleen extracts of ABX chickens was also significantly reduced, including *MDA5*, *OASL*, *Mx*, and *IFITM5* (Fig. 4b), indicating that the innate immune response mediated by IFN-I is impaired.

We investigated whether IFN-I responses driven by commensal microbiota are crucial for antiviral innate immunity. SPF chickens were injected with IFN-I receptor (IFNAR) antibodies to block the IFNA signaling pathway, which was used to validate the effects of microbiota on IFN-I after IBV infection. Compared with that in the PBS control, the secretion of IFN-β in the serum of infected chickens was significantly reduced in ABX and SPF chickens treated with IFNAR antibodies (Fig. 4c). Correspondingly, the difference in viral load between ABX and SPF chickens in the blood or kidney was also significantly increased (Fig. 4d). In summary, the IFN-I amplification pathway related to the intestinal microbiota of SPF chickens is necessary for preventing IBV infection. However, owing to the limitations of chicken research technology, the chicken anti-IFNAR antibody did not completely block the IFNAR pathway. Therefore, IFN-I expression was not completely suppressed.

### *Lactobacillus murinus* promotes macrophage activation and IFN-I responses to IBV infection

**Fig. 5:**
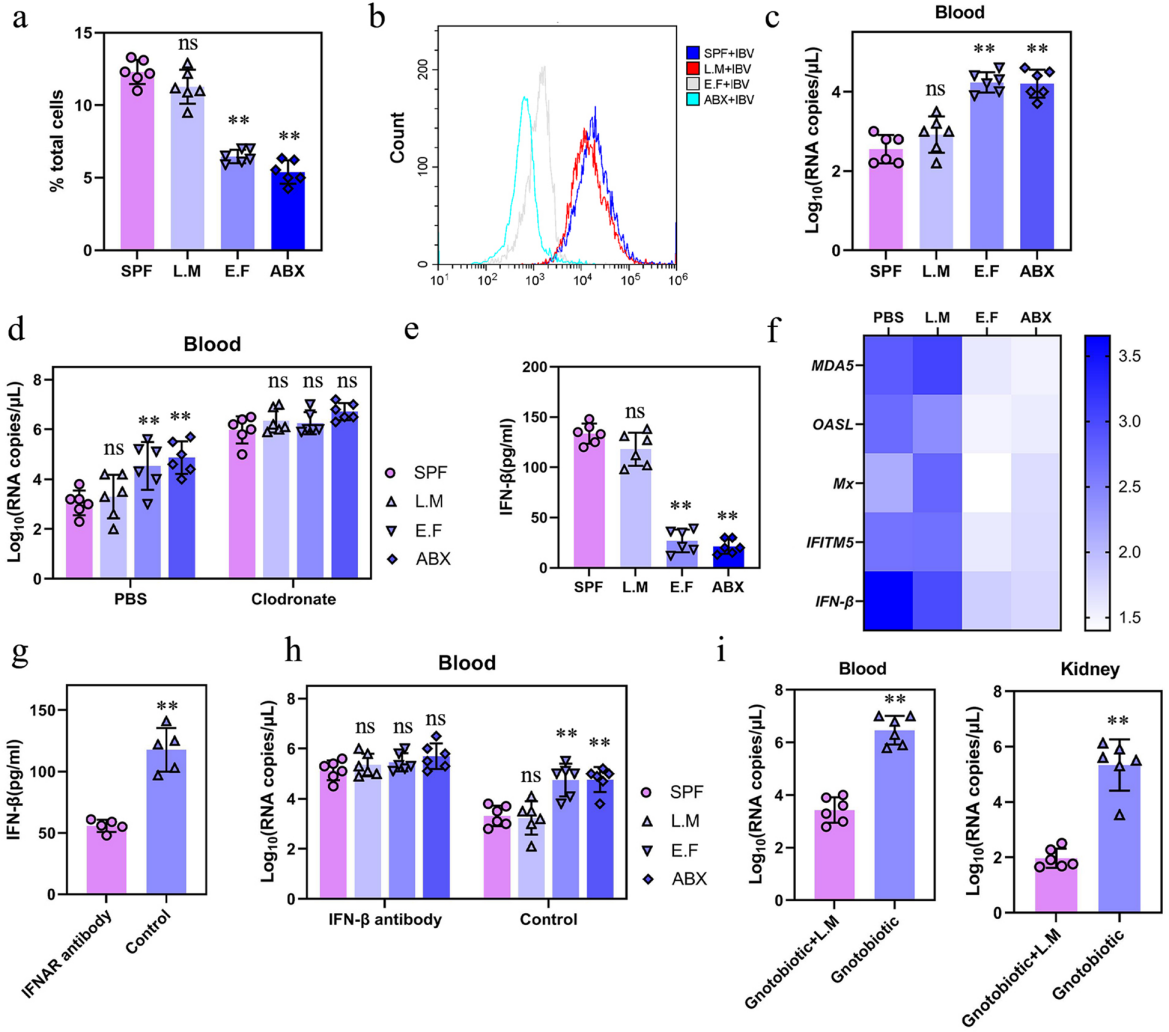
*L. murinus* promotes macrophage activation and IFN-I responses to IBV infection. **a** Frequency of a panel of macrophages in PBMC of mock or infected SPF or ABX chicken (*n* = 6), gavaged with *L. murinus* at 5dpi following IBV inoculation. **b** Expression of MHC-II on PBMC macrophages from mock or infected SPF or ABX chicken, gavaged with *L. murinus* or control at 5 dpi following IBV inoculation. **c** Peripheral blood of ABX chicken (*n* = 6) gavaged with *Lactobacillus* was harvested and tested for viral loads at 5 dpi using qRT-PCR. **d** Viral loads of peripheral blood of infected *L. murinus*-gavaged ABX chicken (*n*= 6) injected with clodronate liposomes. **e** IFN-β levels in the serum of ABX chicken gavaged with *L. murinus* gavaged at 5 dpi following IBV infection as detected by ELISA. **f** Fold change of IFN-β and ISG expression in PBMC and the spleen of SPF or ABX chicken (*n* = 6) gavaged with *L. murinus* at 5 dpi following IBV infection relative to respective mock controls. **g** IFN-β levels in the serum of ABX chicken gavaged with *L. murinus* ABX chicken (*n* = 6) injected with IFNAR antibody. **h** Viral loads of peripheral blood of ABX chicken (*n* = 6) gavaged with *L. murinus* injected with IFNAR antibody. **i** Viral loads of peripheral blood or kidney of gnotobiotic chicken (*n* = 6) gavaged with *L. murinus* injected with IFNAR antibody. Data are from three independent experiments. Biological replicates for each sample are three. *P* values were determined using a one-way analysis of variance; ns, not significant; **P < 0.01

Among the millions of commensal bacteria in the chicken intestine, *Lactobacillus* spp*.* are one of the most abundant in different locations in the intestine [32]. *Lactobacillus* spp. interact with chicken macrophages to induce an antiviral innate immune response [33]. To explore the intestinal-specific strains that resist IBV infection, 16S rRNA sequencing of the feces of surviving and dead SPF chickens infected with IBV was performed, indicating that *Lactobacillus* was the most critical differential bacterium in the intestinal microbiota of surviving and dead chickens. Therefore, it was necessary to investigate whether *Lactobacillus* in SPF chickens modulates IFN-I in macrophages to promote IBV resistance. *Lactobacillus murinus* (*L. murinus*) and *Enterococcus faecalis* (*E. faecalis*) were isolated from the feces of SPF chickens in our laboratory, which have a high abundance of bacteria in the intestines of SPF chickens (Additional file 2). The present study investigated *L. murinus*, which has been shown to have immunomodulatory properties [34, 35]. On the seventh day after ABX treatment, the chickens were gavaged with 100 μL (approximately 10^7^–10^8^ colony-forming units [CFU] per chicken) of *L. murinus* and *E. faecalis* every day. At 5 dpi, we analyzed whether *L. murinus* were colonized, evaluated IBV infection, and calculated the frequency of macrophages and the expression of MHC-II in macrophages of PBMC. Although *L. murinus* cannot colonize the intestinal tract of ABX chickens, it significantly increased the frequency of macrophages (Fig. 5a) and the number of macrophages MHC-II in PBMC (Fig. 5b); however, it was still slightly lower than that of SPF chickens.

In addition, IBV in the blood of the ABX chickens treated with *L. murinus* was significantly reduced compared with that in the blood of the ABX chickens treated with *E. faecalis* and untreated ABX chickens. However, the viral load in the blood of chickens was still higher than that in SPF chickens (Fig. 5c). This indicates that immune regulation to inhibit IBV may be regulated by *L. murinus* and may depend on other commensal bacteria. Similarly, although *L. murinus* could not colonize the intestinal tract of sterile chickens, it could provide protection against IBV infection.

To determine whether *L. murinus* mediates protection against IBV infection through a macrophage-dependent mechanism, ABX chickens gavaged with *L. murinus* were depleted of macrophages and infected with IBV. The results showed that clodronate liposome treatment eliminated the protection against IBV mediated by *L. murinus*, demonstrating that *L. murinus* is necessary for macrophage-mediated protection against IBV infection (Fig. 5d).

Serum was collected at 5 dpi from ABX chickens treated with *L. murinus* to determine whether ABX chickens gavaged with *L. murinus* could recover the IFN-I response to viral infection. The secretion of IFN-β (Fig. 5e) and expression of ISGs in PBMC (Fig. 5f) were analyzed. Compared with the control bacteria (*E. faecalis*), both the secretion of IFN-β and the expression of ISGs in ABX chickens treated with *L. murinus* were significantly restored, although they were still lower than the levels in SPF chickens, indicating that other commensal bacterial species could also induce IFN-I responses in circulating macrophages.

It has been proved that the IFN-I amplification pathway driven by the intestinal microbes of SPF chickens is necessary for resistance to IBV infection and that *L. murinus* mediates protection against IBV infection in an IFN-I-dependent manner. Before IBV inoculation, ABX chickens gavaged with *L. murinus* were treated with anti-IFNAR antibody. *L. murinus* could not increase the secretion level of induced IFN-β (Fig. 5g), nor could it provide resistance to IBV infection (Fig. 5h). Overall, these data indicate that protecting *L. murinus* against viral infection requires macrophages and IFN-I signaling pathways.

Consistent with these results, continuous intragastric gavage of gnotobiotic chickens with *L. murinus* also significantly reduced IBV load in the blood and kidney (Fig. 5i).

### *Lactobacillus murinus*-derived metabolite exopolysaccharide induces IFN-β expression in macrophages to resistance to IBV infection

**Fig. 6:**
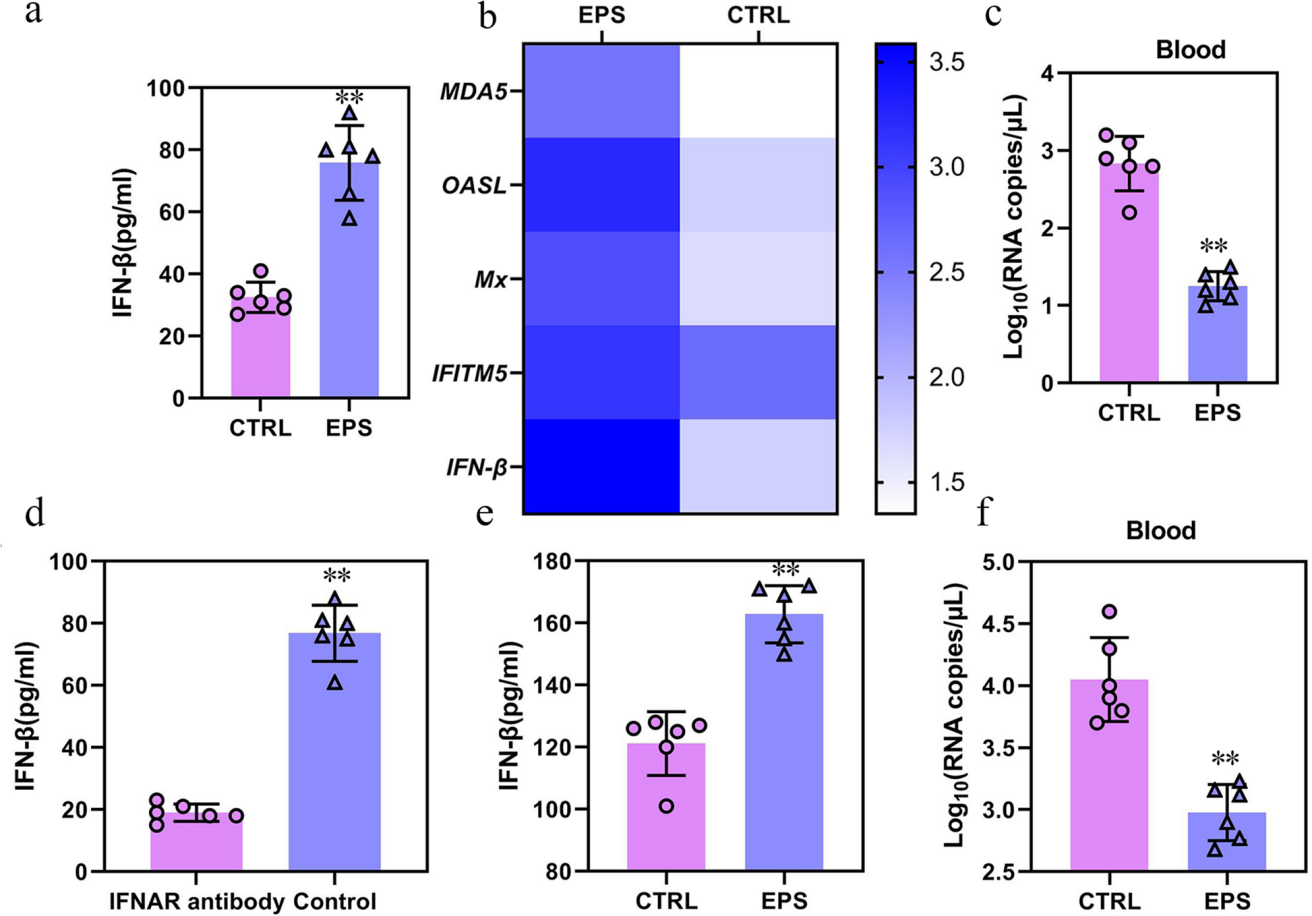
Exopolysaccharide induces IFN-β expression in macrophages to resistance to IBV infection. **a** IFN-β levels in the monocytes/macrophages supernatant from PBMC of ABX chickens (*n* = 6), treated with 50 mg/mL EPS complex and CTRL in vitro. **b** Fold change of IFN-β and ISG expression in PBMC of ABX chickens (*n* = 6), treated with 50 mg/mL EPS following IBV infection relative to respective mock controls. **c** IBV loads of macrophages from PBMC of ABX chickens (*n* = 6), treated with 50 mg/mL EPS complex. **d** IFN-β levels in the monocytes/macrophages supernatant from PBMC of ABX chicken (*n* = 6) injected with IFNAR antibody. **e** IFN-β levels in the serum of ABX chicken (*n* = 6) gavaged with EPS orally starting 7 days prior to and continuing to the day of infection with IBV. **f** Viral loads of peripheral blood of ABX chicken (*n* = 6) gavaged with EPS orally starting 7 days prior to and continuing to the day of infection with IBV. Data are from three independent experiments. Biological replicates for each sample are three. *P* values were determined using a one-way analysis of variance; ns, not significant; **P < 0.01

Exopolysaccharide (EPS) is one of the main secondary metabolites of *Lactobacillus* and has biological functions, such as anti-tumor, anti-oxidation, anti-viral, and immune regulation [36]. EPS produced by specific *Lactobacillus* strains can induce IFN-β and enhance the expression of antiviral factors *RNase L* and *Mx1* in porcine intestinal epithelial cells [37, 38]. To explore whether EPS of *L. murinus* induced IFN-β to resist IBV, monocytes and macrophages from PBMC of ABX chickens were isolated, treated with 50 mg/mL EPS complex and vehicle control (CTRL) for 24 h, and then infected with IBV. The secretion of IFN-β in the cell supernatant was analyzed. The data showed that EPS significantly induced the secretion of IFN-β (Fig. 6a) and ISGs from PBMC (Fig. 6b), while IBV loads were significantly reduced (Fig. 6c). Monocytes/macrophages were isolated from PBMC of ABX chickens treated with IFNAR antibody, treated with EPS, infected with IBV, and analyzed for IFN-β secretion. The results showed that IFN-β signaling through IFNAR was necessary for inducing IFN-β secretion in EPS-treated cells (Fig. 6d). These data indicate that EPS induces IFN-β secretion in SPF chicken monocytes/macrophages during IBV infection in vitro.

To validate the effect of EPS in vivo, we analyzed the expression of IFN-β in macrophages after SPF chickens were fed 150 mg EPS orally starting 7 days prior to and continuing to the day of infection with IBV. Compared with that in the CTRL, secretion of IFN-β significantly increased after oral gavage administration of EPS (Fig. 6e) and the IBV load decreased (Fig. 6f), indicating that EPS can induce IFN-β in SPF chicken monocytes/macrophages, thereby causing resistance to IBV infection.

### IFN-β induction is a shared function of lactobacillus spp. metabolites EPS

**Fig. 7:**
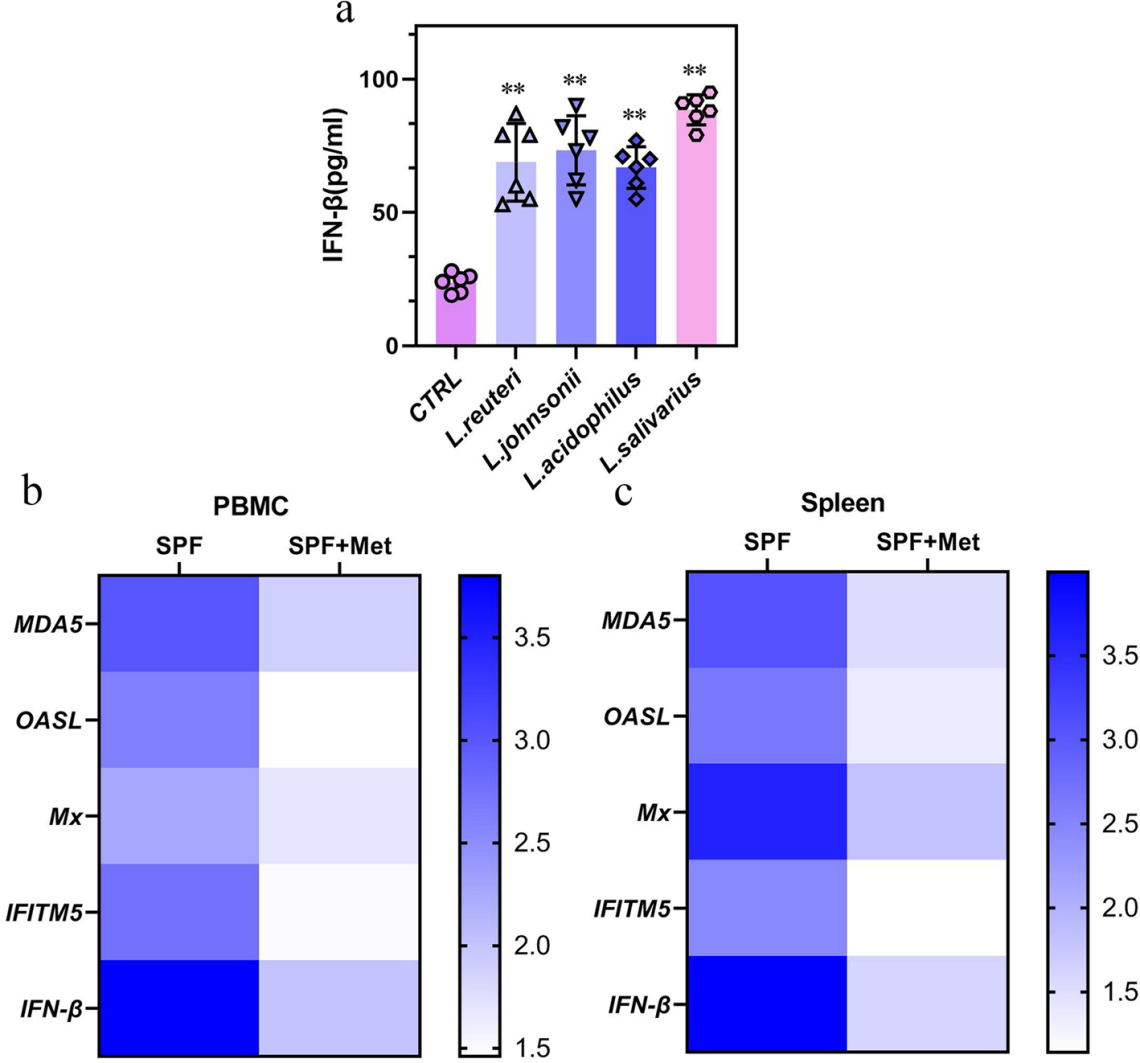
IFN-β induction is a shared function of *Lactobacillus* spp*.* metabolites EPS. **a** Macrophages were differentiated from SPF chicken (*n* = 6) or ABX chicken (*n* = 5) mice and treated with 50 mg/mL EPS for 6 h. IFN-β in the supernatants was measured by ELISA and normalized by subtracting vehicle control. **b**, **c** qRT-PCR analysis of ISG expression was performed on the PBMC and spleen harvested from SPF chicken (*n* = 6), SPF chicken after 2 weeks of metronidazole (Met) treatment. Fold change gene expression in the PBMC (**b**) or spleen (**c**) was calculated compared to SPF chicken. Data are from three independent experiments. Biological replicates for each sample are three. *P* values were determined using a one-way analysis of variance; ns, not significant; **P < 0.01

EPSs are not unique to *L. murinus*, and the induction of IFN-β by *Lactobacillus* spp*.* represents a broader mechanism by which commensal microbes are capable of influencing the host immune system. EPSs were isolated from several different species of *Lactobacillu*s to test their effects on cytokine secretion by monocytes/macrophages. Treatment with exopolysaccharides extracted from all *Lactobacillus* spp*.* metabolites induced IFN-β expression in macrophages (Fig. 7a), indicating that *Lactobacillus* spp*.* EPSs induce IFN-β expression. To address the role of *Lactobacillus* spp*.* in regulating the IFN-I response in vivo, ISG expression was compared in SPF chickens and SPF chickens 7 days post-treatment with metronidazole before IBV infection, a bactericidal antibiotic that specifically targets anaerobic bacteria, including *Lactobacillus* spp*.* SPF chickens treated with metronidazole alone exhibited significant decreases in ISG expression in PBMC (Fig. 7b) and the spleen (Fig. 7c). These findings confirm the potential of anaerobic commensal bacteria, of which *Lactobacillus* spp*.* are one of the most dominant taxa, to regulate the host IFN-I response to resist IBV infections.

## Discussion

In this study, we found that deletion of the intestinal microbiota can exacerbate nephropathogenic IBV infection in SPF chickens. Consistent with the ABX chickens’ phenotype, viral loads of IBV in the peripheral blood and kidneys of gnotobiotic chickens increased. Additionally, ABX chickens showed a decrease in the number and activity of macrophages after IBV infection, which was associated with a decrease in systemic ISG expression. ABX chickens gavaged with *Lactobacillus* could activate macrophages through its metabolite extracellular EPS to repair the immune damage caused by the deletion of intestinal microbiota, and induce the expression of IFN-β and ISGs to resist IBV infection.

The microbiota diversity of chickens is relatively lower than that of the mammalian gut microbiota. The chicken gut microbiome is a diverse and complex ecosystem, including a large number of bacteria [39], which can metabolize food components [40, 41], support the development of immune function [42], and confer resistance to pathogenic infections [43, 44].

The ABX cocktail treatment method for the depletion of intestinal microbiota has been widely used in mammalian studies, and there have been many studies on its application in chickens and ducks. Corroborating the existing literature, our experiments show that ABX treatment can eliminate most of the bacteria in SPF chickens’ fresh feces, while promoting an increase in the abundance of conditional pathogens *Escherichia*, *Shigella*, and *Klebsiella*. Therefore, we established a gnotobiotic chicken IBV infection model and ruled out the possibility that an increase in pathogenic bacteria causes a decrease in the host’s resistance to IBV infection.

Macrophages are important immune cells that are resistant to microbial infections and participate in the innate immune response and subsequently acquired immunity. IFN-I is a core effector cytokine of host antiviral natural immunity. Reddy et al. [28] showed that the Belgian nephrogenic IBV strain B1648 could infect blood monocytes, which may promote the spread of IBV to the internal organs, kidneys, liver, spleen, and lungs. Our data showed that the intestinal microbiota of SPF chickens antagonizes nephropathogenic IBV infection by regulating the activity of monocytes/macrophages and IFN-I responses. Two authoritative studies have shown that microbiota constitutively induces the production and basic level of ISG expression in pDC at the type I IFN system locus [25, 26]. Zhu et al. have demonstrated that the intestinal microbiome protects against systemic enteric virus infection by activating macrophages and type I IFN responses. They believed that the signal of the symbiotic bacteria drives the very rapid first wave of IFN-I (especially IFN-β) production in the stable pDC to activate the NK cells, macrophages, and overall innate immune response to viral infection [24]. However, whether SPF chicken pDC secrete type I IFN during IBV infection requires further investigation.

Research on intestinal microbiota has entered the “post-sequencing” era, and functional studies of specific intestinal bacteria have gradually become mainstream. Steed et al. [45] reported that intestinal *Clostridium orbiscindens* can produce metabolic desaminotyrosine, which upregulates the IFN-I signaling pathway and downstream ISG expression in pulmonary macrophages and enhances innate immune responses to resist AIV. Zhu et al. [24] identified a commensal bacterial strain, *B. coccoides*, that can activate IFN-I and ISG responses in macrophages through IFNAR- and STAT1-mediated signaling pathways, thereby limiting encephalomyocarditis virus replication and neuropathogenesis. In the past 10 years, different *Lactobacillus* and other probiotics have been shown to interact with the mucosal immune system cells to regulate the immune system of chickens [46–48]. It has been reported that *L. acidophilus*, *L. reuteri*, and *L. salivarius*, when used alone or in combination, induce the antiviral response via chicken macrophages to avian influenza virus [32]. In this study, *Lactobacillus* was the most critical differential bacterium in the intestinal microbiota of surviving and dead chickens infected with IBV. In addition, *L. murinus* was one of the most prolific *Lactobacillus* spp. in the intestine of SPF chickens. Continuous gavaging with *L. murinus* can activate IFN-I and ISG responses in monocytes/macrophages through IFNAR-mediated signaling pathways in chickens, thereby limiting IBV infection. It was unexpected that exogenous *Lactobacillus* spp., including *L. murinus* isolates, would not effectively mono-colonize either ABX chickens or gnotobiotic chicken models. Consistent with our findings, Kubasova et al. [34] found that none of the gram-positive isolates colonized the chicken cecum during the first week of life. However, gram-negative bacteria can colonize the chicken cecum independently, and their colonization has little effect on the colonization of other microbiota members. They believed that gram-positive bacteria seem less suitable for single-dose chicken probiotic products because these products do not colonize effectively. Clavel et al. [49] also confirmed that *Anaerotignum lactatifermentans* and *Limosilactobacillus oris* were not detected after synthetic bacterial community intervention in newly hatched chickens, and other *Lactobacilli* were only present at low relative abundances. Although gram-negative isolates may successfully colonize chicken ceca and do not require co-colonization by any other members of the microbiota, it cannot be ruled out that gram-positive bacterial colonization of the intestines of ABX- or germ-free chickens requires co-colonization of additional bacterial members. Given the harsh environment of the chicken proventriculus/gizzard (pH 1.2), losses may occur during the transformation of isolates, as described for bacterial strains ingested by humans [50]. Furthermore, in the animal intestine, the microbiota is known to develop from an aerobic/facultative anaerobic state to an obligate anaerobic state; thus, even in repeated gavage, increasing the frequency of intervention to ensure engraftment of strictly anaerobic species may be useful [51].

**Fig. 8:**
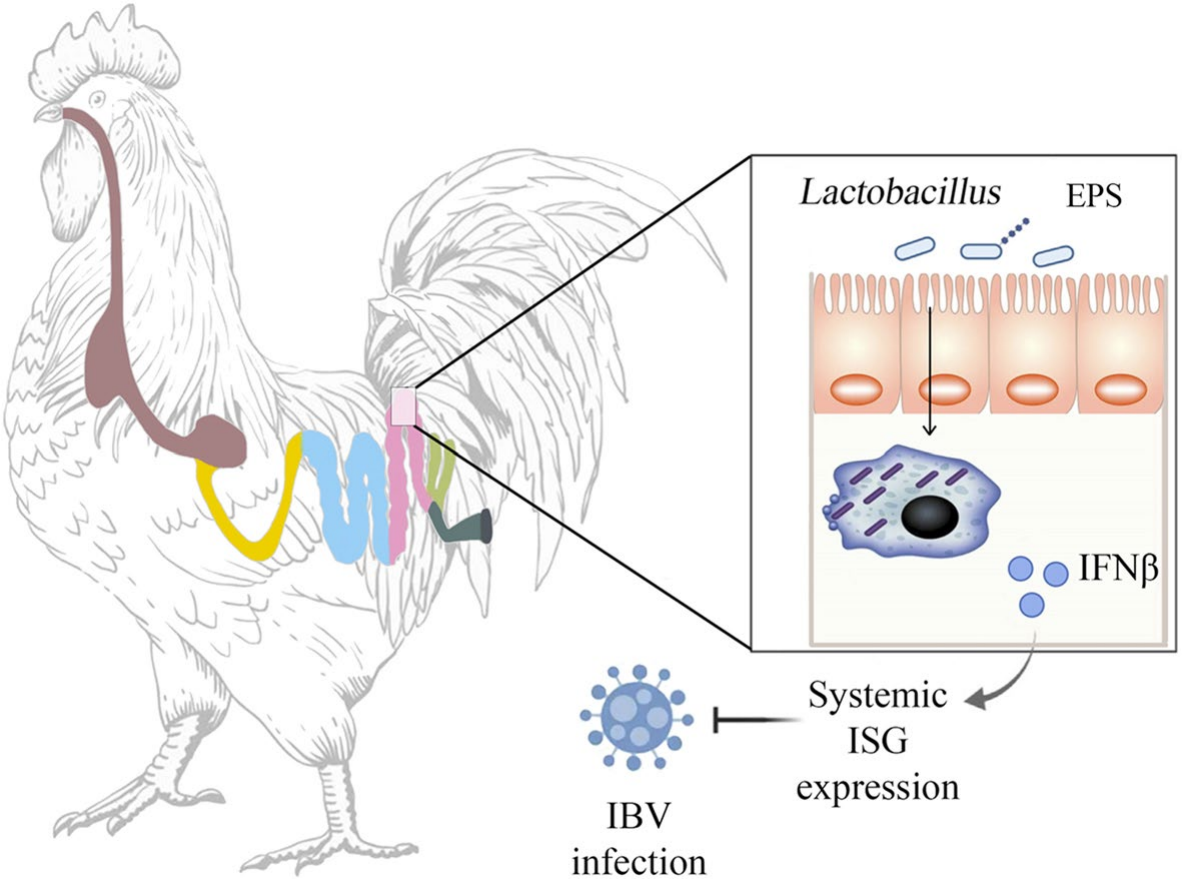
Schematic summary: EPS from *Lactobacillus* of SPF chicken intestinal microbes can activate macrophage to secrete IFN-β, engine subsequently systemic ISG expression to resistance to nephropathogenic IBV infection

Interestingly, *Lactobacillus* spp*.* metabolites have been shown to have antiviral activity and may represent a promising method for the treatment of viral diseases [52, 53]. This study showed that EPS induces IFN-β secretion in SPF chicken monocytes/macrophages to limit IBV infection (Fig. 8). However, the mechanism by which EPS induces IFN-β secretion requires further investigation.

## Conclusions

This study revealed the immune mechanism of the SPF chicken intestinal microbiota against nephropathogenic IBV infection. In addition, we determined that although *Lactobacillus* isolates cannot be colonized effectively, continuous gavaging with it can activate IFN-I and ISG responses in monocytes/macrophages through IFNAR-mediated signaling pathways in chicken, thereby limiting IBV infection.

## Materials and methods

### Cell, bacteria isolates, virus, and EPS purification

The TW I-type IBV strain was maintained at the Animal Immunology Laboratory of the College of Life Science and Agriculture Forestry, Qiqihar University. The virus was propagated in SPF chicken embryos at 9–11 days old.

Blood from SPF chickens was collected in a tube containing 10 U/mL heparin (Ncbiotech Co., Ltd.) by cardiac puncture. PBMCs were isolated using an avian lymphocyte isolation kit (Tianjin Haoyang Biological Technology), according to the manufacturer’s instructions.

Splenocytes were isolated according to a previously reported method [54]. The chicken spleen was removed, cut into small pieces, placed in ice-cold Hank’s Balanced Salt Solution (HBSS) without calcium and magnesium, and 10 mM HEPES 0.45% (w/v) glucose and 1 mM sodium pyruvate) using a borosilicate glass homogenizer (Fisher Scientific UK Ltd.) for homogenization, and then filtered through a 100-μm cell strainer. Homogenized solution for birds older than 3 weeks was overlaid on Histopaque-1·077 (Sigma-Aldrich) and centrifuged at 400 × *g* for 20 min with the brake off. The cells at the density interface were collected, washed, and resuspended in PBS supplemented with 2% FBS and 1 mM EDTA.

Monocytes/macrophages were sorted and recycled from freshly isolated chicken PBMC or splenocytes using mouse anti-chicken monocyte/macrophage-PE clone KUL01; cultured in a complete medium supplemented with 10% fetal bovine serum (FBS), 100 units/mL penicillin, 100 μg/mL streptomycin, and RPMI 1640; and stored at 41°C and 5% CO_2_.

The species samples of *L. reuteri*, *L. murinus*, *L. johnsonii*, *L. acidophilus*, *L. salivarius*, and *E. faecalis* were isolated and stored in the laboratory. *Lactobacillus* spp*.* were cultured in MRS medium at 37 °C, whereas *E. faecalis* was cultured in LB medium at 37 °C.

EPS from *Lactobacillus* spp*.* was extracted and fractionated according to the method described by Lü et al. [55]. The bacteria were incubated at 35 °C for 28 h and centrifuged (6000 rpm, 20 min) to separate the cells. The supernatant was concentrated by rotary evaporation, and the protein was collected. Three times the volume of ethanol was added to the culture supernatant to separate the EPS at 4 °C for 12 h. The precipitate was dissolved in distilled water, dialyzed with dH_2_O (Mw cut-off value: 8000–14000 Da), and freeze-dried to obtain a dry EPS powder. Subsequently, the powder was re-dissolved in distilled water, decolorized with D4020 macroporous resin, and finally collected as crude EPS. The sample (400 mg) was loaded onto a DEAE-Sepharose Fast Flow column (ø 2.6 cm × 1 m) and eluted with different concentrations of NaCl (0, 0.1, 0.3, 0.5, 0.7 mol/L). NaCl was dissolved in PBS (pH 6) at a flow rate of 1 mL min^−1^. The main part (30 mg) obtained was passed through a Sephacryl S-300 column (ø 2.6 cm × 1 m), and ultrapure water with a flow rate of 0.5 mL/min was used. The purified fraction was used for further experiments.

### Experimental animals and chicken embryos

One-day-old SPF female chickens were obtained from the Laboratory Animal Technology Department of Harbin Veken Biotechnology Development Company. The 9–11-day-old SPF chicken embryos were purchased from the Laboratory Animal Technology Department of Harbin Veken Biotechnology Development Company.

The gnotobiotic chickens were bred according to a previously described protocol [56] with slight modifications. SPF chicken embryos were treated with sporicidin disinfectant solution (Contec, Inc.) and incubated at 37 °C and 55% humidity (pre-treated with sporicidin). Potassium permanganate aqueous solution (1% [wt/vol]) was used to maintain humidity. Nineteen-day-incubated live eggs were transferred to a biological safety cabinet, soaked in sporicidin solution for 15 s, and then wiped with a sterile cloth soaked in sterile water. The eggs were sprayed with 5% peracetic acid, exposed for 20 min, and then transferred to the isolator. The eggs were maintained at 37 °C and 65% humidity until hatching on day 21. After hatching, chickens were provided with sterile water and gamma-ray irradiated starting diets to meet the nutritional needs of the chicks and were monitored every day. To assess the sterility of the isolator, swabs were collected from eggshells, feces, and the bottom of the isolator on the second day after hatching; transferred to the anaerobic transport medium; placed in BHI-M agar streak plates; and incubated aerobically at 37 °C. The plates were checked for bacterial colonies after 24 and 48 h of incubation.

### Antibiotic treatment

The 7-day-old SPF chickens were treated with ABX in drinking water. ABX was administered at the following doses: vancomycin (80 mg/kg), neomycin (300 mg/kg), metronidazole (200 mg/kg), ampicillin (0.2 mg/kg), and colistin (24 mg/kg). ABX-treated chickens also received 2 mg/kg amphotericin B daily to prevent fungal overgrowth [4, 7, 27, 57]. Water was changed every 2 days, and ABX treatment was started 2 weeks before infection.

### IBV infection

The Reed–Muench method was used to determine 50% of the chicken embryo infection dose (EID_50_)/mL in 9–11-day-old SPF chicken embryos. Twenty-one day-old SPF chickens were infected with 0.2 mL 10^6^ EID_50_/0.1 mL IBV via the oculonasal route. Chickens in the uninfected group were treated with equal amounts of allantoic fluid collected from uninfected chicken embryos. They were housed in negative-pressure isolation hoods to provide sufficient sterile feed and drinking water, and clinical signs were observed and recorded. The chickens were euthanized and necropsy was performed at the indicated time. Tracheal, kidney, and blood samples were collected for histopathological analysis, immunohistochemistry, and virus quantitative analysis.

### Bacteria depletion analysis

In the appropriate amount of fresh feces of ABX chicken and SPF chicken, SSL buffer was added and mixed thoroughly, and DNA was extracted from intestinal bacteria using Hi-Pure Stool DNA Kits (Qiagen). Amplification with a PCR kit amplified the V3+V4 region of the 16S rRNA gene. The PCR products were purified using the AMPure XP Beads kit (Qiagen), quantified using the ABI Step One Plus Real-Time PCR System, and then pooled and sequenced in the PE250 mode of the Hiseq2500 system. QIIME (version 2) and SPSS software (version 23.0) were used for alpha diversity analysis of duck intestinal flora, and beta diversity analysis was performed using Muscle (v3.8.31) and TreeBeST (v1.9.2) software. The RDP classifier software performs annotation classification of the intestinal flora species, and the LEfSe software analyzes the species differences in the intestinal microbiota.

Homogenization of fecal samples collected from chickens with depleted microbiota on day 7 after ABX treatment was spread on brain heart infusion (BHI) agar containing 10% sheep blood and incubated at 37 °C under anaerobic conditions for 2 days, and then incubated at 37 °C under aerobic conditions for 1 day to confirm effective microbial consumption.

### Antibiotic treatment and infection of chicken embryo

Eleven-day-old chicken embryos were injected with ABX into the allantoic fluid, and the total concentration was as follows [27]: vancomycin (500 mg/L), neomycin (500 mg/L), metronidazole (500 mg/L), ampicillin (1 g/L), colistin (80 mg/L), or sterile PBS. After incubation at 37 °C for 24 h, chicken embryos treated with ABX cocktail or PBS were injected with 100 μL of 10^5^ EID_50_ of IBV. The allantoic fluid was collected 72 h after infection for quantitative analysis of IBV.

### RNA extraction and cDNA synthesis

In a 10-cm^2^ culture area of adherent cells or 50–100-mg animal tissue (ground into powder in liquid nitrogen) or 500 μL chicken embryo allantoic fluid, 1 mL TRIzol reagent (Invitrogen) was added depending on the material, and the lysis time varied on ice. After extraction with an RNA isolator, the aqueous phase was transferred to the RNA extraction column and processed following the manufacturer’s instructions. A total of 500 ng of total RNA was synthesized by reverse transcription using oligo (dT)_18_ (0.25μg), random primers (0.1 μg), and MC5-reverse transcriptase (Yemico Enzyme Biotech).

### Histopathological analysis and immunohistochemistry

All animals underwent complete postmortem inspection. Tissue samples from the trachea (one proximal cross-section and the other end cross-section) and kidney were preserved in 10% neutral formalin. After fixation, the tissues were treated with paraffin blocks, 4-μm sections, and stained with hematoxylin and eosin for microscopic examination. A certified veterinary pathologist was blinded to the experimental conditions, and the lesion was evaluated histologically. Lesion intensity was graded as follows: 0, no lesion; 1, smallest; 2, mild; 3, moderate, marked, or severe.

Mouse anti-IBV N protein was used as the antibody (antibody dilution 1/500, 4 °C 16–18 h). Horseradish peroxidase-labeled goat anti-mouse secondary antibody was used for immunohistochemical staining (HRP; Dako, LSAB2 system-HRP, K0675) (Thermo Scientific) ( antibody dilution 1/100, 37°C 2 h). The negative control consisted of sections incubated with non-specific primary antibodies.

### Phospholiposomes and anti-IFNAR antibody treatment

To deplete monocytes/macrophages, each chicken was intraperitoneally injected with 500 μL chlorophosphate liposome suspension (Free University Amsterdam) and 500 μL control liposomes (Free University of Amsterdam), according to the manufacturer’s instructions, 2 days prior to ABX treatment. Mouse anti-chicken monocyte/macrophage-PE-KUL01 was used to detect chicken splenocytes and analyze the efficiency of clodronate liposomes in eliminating monocytes/macrophages.

To block the IFN signaling pathway, each chicken was intraperitoneally injected with PBS (control) and an anti-IFNAR antibody (Abcam) at a dose of 500 μg for 2 days prior to ABX treatment.

### Single-strain treatment

On the seventh day after ABX treatment, the chickens were gavaged with 100 μL (approximately 10^7^–10^8^ CFU per chicken) of *Lactobacillus* spp*.* or *E. faecalis* every day until 5 dpi. Fecal samples were collected 48 h after bacterial treatment to determine the colonization efficiency.

### Flow cytometry and fluorescence-activated cell sorting (FACS)

SPF chicken PBMC and splenic monocytes/macrophages were identified as the mouse anti-chicken monocyte/macrophage-PE clone KUL01 (Southern Biotechnology Associates). Monocytes/macrophages were sorted and recycled from freshly isolated chicken PBMCs using mouse anti-chicken monocyte/macrophage-PE clone KUL01. The surface molecule reflecting chicken macrophage activation was MHC-II (Southern Biotechnology Associates). Activated macrophages were identified as KUL01 and MHC-II double positive.

### Virus detection and quantification

IBV RNA was extracted from cells or tissues using TRIzol reagent (Invitrogen), according to the manufacturer’s instructions. Using the PrimeScript™ RT reagent Kit with gDNA Eraser (Perfect Real Time) (Takara Biomedical Technology Co.), 1 μg of total RNA from each sample was reverse transcribed into cDNA. The IBV N gene was used to determine the IBV copy number using a fluorescence quantitative PCR reagent (Beijing Biotech Company). Specific primers were designed according to the IBV N gene (accession number: FJ904723.1), as shown in Table 1. The fluorescent probe sequence is 5′-FAM-CACCACCAGAACCTGTCACCTC-BHQ1-3′. The thermal cycling steps were as follows: 94 °C, denaturation for 2 min, 40 cycles, and PCR cycle (94 °C, 15 s; 60 °C, 30 s).

**Table 1:** Primers used for qRT-PCR

Gene	Primer sequence (5′–3′)	
	Forward primer	Reverse primer
*MDA5*	F: TCAGGAGGAGGACGACCACGAT	R: TTCCCACGACTCTCAATAACAG
*OASL*	F: CTTGACAGTGGAGAGGG	R:ACGAAGACCTGGATCTCGGA
*Mx*	F:AGGGGCCATCACATTCACAT	R:AGATACTTCAGGGGATTCTC
*IFITM5*	F:AAGGTGTCGGAGGATGGTGGTC	R: GGAATCAGCCGCTTGAGACGAG
*IFN-β*	F:GCCCACACACTCCAAAACACTG	R: TTGATGCTGAGGTGAGCGTTG
*IBV-N*	F: CAAGCTAGGTTTAAGCCAGGT	R: TCTGAAAACCGTAGCGGATAT
*β-actin*	F: ATTGCTGCGCTCGTTGTT	R:ATCGTACTCCTGCTTGCTGATCC

### IFN-β ELISA analysis

SPF or ABX chickens were euthanized for serum analysis, and blood was collected in 1.1-mL Z-Gel microtubes (Salstette). The blood was placed at room temperature for 30 min and centrifuged to remove coagulated cells (12,000 rpm, 10 min), and an IFN-β ELISA Kit (USCN Life Science) was used to quantify the IFN-β level in the serum according to the manufacturer’s instructions. For cell culture supernatant analysis, cell-free supernatants were collected, and cell culture and IFN-β levels were quantified according to the manufacturer’s protocol.

### qRT-PCR analysis of gene expression

The PrimeScript™RT kit and gDNA Eraser were used to extract total RNA from tissue and reverse transcribed into cDNA, which was then used to detect the relative gene expression of IFN-β and ISGs (*MDA5*, *OASL*, *Mx*, *IFITM5*). The primers used are listed in Table 1. β-actin was selected as the reference gene. The thermal cycling conditions were as follows: 94 °C for 2 min, followed by 40 PCR cycles (94 °C, 15 s; 60 °C, 30 s). The final concentration of each primer is 1 μM. qPCR was performed using a LightCycler 96 (Roche). The 2^-△△CT^ method was used to calculate the fold change in gene expression.

### Statistical analysis

GraphPad Prism software (version 9.0) was used for all statistical analyses. Error bars represent the standard deviation of the mean in all figures. The *P-*value in two-group data comparisons was determined using an unpaired two-tailed Student’s *t* test; the *P*-value in multiple comparisons was determined using a one-way analysis of variance.

## Supplementary Information

**Additional file 1: Figure S1.** The dilution curve that was generated based on an operational taxonomic unit (OTU) level indicated that the sampling work had sufficient sequences to analyze bacterial diversity.

**Additional file 2: Figure S2.** Species level community composition of intestinal microbiota of specific pathogen-free chickens.

## Data Availability

The raw reads were deposited into the NCBI Sequence Read Archive (SRA) (BioProject ID: PRJNA844284). All data were available upon request from the authors.

## Abbreviations

SPF: Specific pathogen free
IBV: Infectious bronchitis virus
ABX: Broad-spectrum antibiotics
AIV: Avian influenza virus
IFN-I: Type I interferon
ISGs: IFN-stimulated genes
pDC: Plasmacytoid dendritic cell
EPSs: Exopolysaccharides
OTU: Operational taxonomic unit
PCoA: Principal coordinate analysis
qRT-PCR: Quantitative reverse transcription-polymerase chain reaction
PBMC: Peripheral blood mononuclear cell
IFNAR: IFN-I receptor
*L*. *murinus*: *Lactobacillus murinus*
*E*. *faecalis*: *Enterococcus faecalis*
CTRL: Vehicle control
FBS: Fetal bovine serum
EID_50_: Chicken embryo infection dose
dpi: Days post-infection
