# Supplementary figures and images for: Chicken intestinal microbiota modulation of resistance to nephropathogenic infectious bronchitis virus infection through IFN-I
Source: Microbiome. 2022 Oct 3;10:162. doi: 10.1186/s40168-022-01348-2 (PMC9527382; doi:10.1186/s40168-022-01348-2)

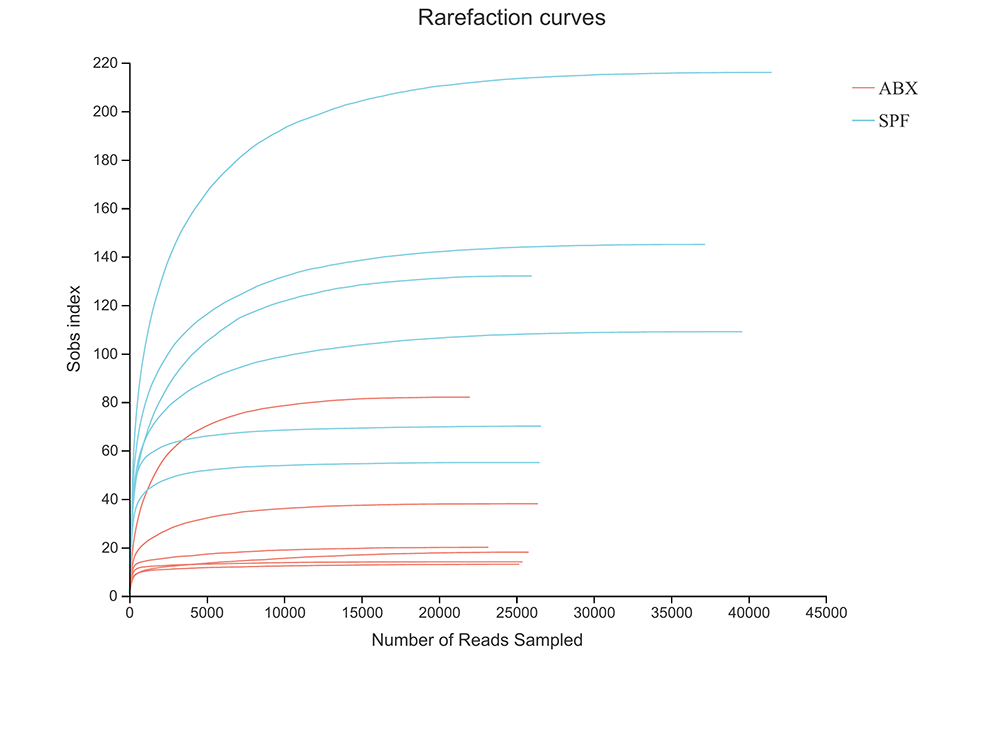

Supplement: Supplementary file 2 — Additional file 1: Figure S1. The dilution curve that was generated based on an operational taxonomic unit (OTU) level indicated that the sampling work had sufficient sequences to analyze bacterial diversity. [file 40168_2022_1348_MOESM1_ESM.tif]

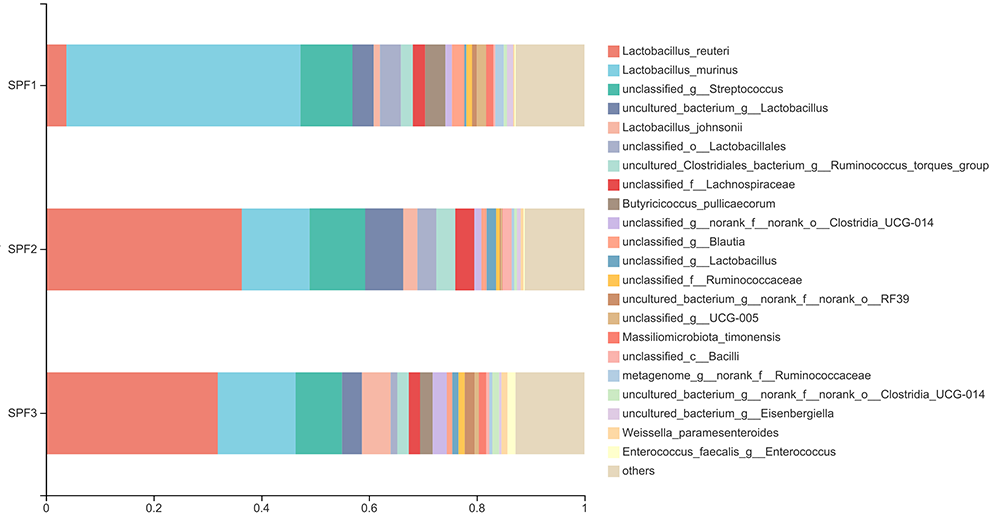

Supplement: Supplementary file 3 — Additional file 2: Figure S2. Species level community composition of intestinal microbiota of specific pathogen-free chickens. [file 40168_2022_1348_MOESM2_ESM.tif]
